# Artificial intelligence-driven prediction and design of cell-penetrating peptides for advanced drug delivery system

**DOI:** 10.3389/fphar.2026.1845913

**Published:** 2026-08-26

**Authors:** Bo Yu, Yue Lu, Zeying Kuang, Mengfan Zhao, Yi Quan, Hongyun Huang

**Affiliations:** 1 Rheumatology and Immunology Department, Affiliated Hospital, Southwest Medical University, Luzhou, China; 2 Sichuan Treatment Center for Gynaecologic and Breast Diseases (Breast Surgery), Affiliated Hospital, Southwest Medical University, Luzhou, Sichuan, China

**Keywords:** cell-penetrating peptides (CPPs), *de novo* peptide design, drug delivery, large language models (LLMs), machine learning (ML), peptide delivery system

## Abstract

**Background:**

Cell-penetrating peptides (CPPs) are promising delivery vectors for transporting therapeutic agents across cellular membranes. However, their rational design remains challenging because the relationship between peptide sequence and translocation efficiency is highly complex and nonlinear.

**Methods:**

In this study, we developed an artificial intelligence-driven framework that integrates biochemical rules derived from large language models (LLMs) with conventional peptide descriptors for CPP prediction and design. Interpretable rules extracted from GPT-4o and DeepSeek were encoded as binary feature vectors and combined with sequence-based descriptors to construct hybrid machine learning models. Model performance was evaluated on the benchmark CPP924 dataset using repeated stratified cross-validation, and the optimized models were further used for *de novo* CPP generation. The resulting candidates were subsequently assessed using multiple established computational benchmarks.

**Results:**

The top-performing hybrid classifier achieved a cross-validated accuracy of 0.91 ± 0.03 (best single held-out split, 0.94) on the CPP924 dataset. The LLM-derived rules outperformed conventional physicochemical and fingerprint descriptors and matched amino-acid composition; integrating the rule and composition features yielded the best overall classifier. Using the optimized RF-GPT-Fre and RF-DS-Fre models, we generated six *de novo* CPP candidates that are sequence-novel (≤53% identity to any training peptide) and retain CPP-like composition and structural features. In-silico evaluation across established tools supports computational prioritisation of these candidates for experimental testing.

**Conclusion:**

These findings demonstrate that combining LLM-derived biochemical knowledge with machine learning improves interpretable CPP prediction and candidate prioritisation. This study provides a reproducible computational strategy for peptide engineering and establishes a basis for the experimental evaluation of next-generation drug-delivery vehicles.

## 1 Introduction

Cell-penetrating peptides (CPPs) are short peptides that uniquely cross cell membranes to enable intracellular delivery of a broad range of therapeutics, from small molecules to nucleic acids, peptides, and nanoparticles (Goddard et al., 2018; Milletti, 2012; Guidotti et al., 2017; Chen et al., 2022). CPPs efficiently cross cellular membranes with minimal cytotoxicity, making them a versatile tool for biomedical applications, including drug delivery, gene therapy, and molecular imaging. (Copolovici et al., 2014).

In drug delivery, CPPs offer a versatile platform to overcome biological barriers that have traditionally limited therapeutic efficacy (Xie et al., 2020). By binding or encapsulating cargo molecules, CPPs can enhance cellular uptake, promote endosomal escape, and improve the bioavailability and efficacy of a wide range of drugs. In particular, the development of CPPs mediated peptide delivery systems has opened up new possibilities for the direct delivery of otherwise impermeable drugs into target cells (Berillo et al., 2021; Tesauro et al., 2019; Kumar Malik et al., 2007). Recent studies have also highlighted significant advances in breast cancer therapy using CPP-based strategies. HER2-targeted CPPs have been designed to deliver genome-editing cargos such as zinc-finger nucleases to disrupt mTOR signaling and overcome trastuzumab resistance (Puria et al., 2012), while other CPP-assisted approaches have enhanced the efficacy of chemotherapeutics including cyclophosphamide, epirubicin and 5-fluorouracil by modulating key apoptotic regulators in breast cancer cells (Chow and Loo, 2003). These findings collectively underscore the emerging therapeutic relevance of CPPs in breast cancer. However, despite their great potential, their rational design and optimization remain extremely challenging. The exact mechanism of their membrane transport is complex and multifactorial, involving not only the peptide sequence motifs but also physicochemical properties such as charge distribution, hydrophobicity, amphiphilicity and secondary structure propensity. Therefore, more efficient and targeted CPPs are urgently needed to ensure therapeutic specificity and minimize off-target effects, which highlights the importance of intelligent design strategies.

The integration of artificial intelligence (AI) and pharmaceutics has greatly accelerated the development of peptide delivery systems and the development of the field of pharmaceutics (Sahu et al., 2022; Vora et al., 2023; Bhattamisra et al., 2023). For example, through multidisciplinary integration, the cell-penetrating peptide called anticancer peptides (ACPs) were designed to be highly specific to cancer cells and encapsulated using nanotechnology, demonstrating therapeutic efficacy against triple-negative breast cancer (Chen et al., 2022). Traditional challenges of peptide delivery, such as poor stability, low membrane permeability and susceptibility to enzymatic degradation, have been increasingly addressed by AI driven approaches. Machine learning (ML) (Jordan and Mitchell, 2015) models, including Support Vector Machine (SVM), Random Forest (RF), and Deep Neural Network (DNN), have been used to predict CPPs based on sequence features such as amino acid composition, net charge, and hydrophobicity (Wei et al., 2019; Arif et al., 2021; Attique et al., 2020; Zhu et al., 2024). More sophisticated methods such as Convolutional Neural Network (CNN) and Graph Neural Network (GNN) are able to model complex sequence-structure-function relationships, thereby facilitating the rational design of peptides with enhanced delivery capabilities (Zhu et al., 2024; Imre et al., 2025). In addition, predictive models for evaluating peptide immunogenicity have been developed, enabling the design of delivery vehicles with lower immune activation (Bryson et al., 2010; De Groot and Moise, 2007). In addition to improving the design of free peptides, AI has also facilitated the creation of peptide drug conjugates (PDCs) and peptide nucleic acid complexes by modeling molecular interactions and predicting delivery effects across different biological barriers, including the blood brain barrier (Cooper et al., 2021; Zhang et al., 2025). Recent advancements in large language models (LLMs) such as GPT-4o (Achiam et al., 2023) and DeepSeek (Liu A. et al., 2024) have further expanded the frontier of artificial intelligence applications in pharmaceutical research (Liu K. et al., 2024). Unlike traditional ML methods that rely heavily on manually curated features, LLMs possess the capacity to infer complex biochemical patterns directly from massive text and sequence data.

The integration of structure prediction platforms such as AlphaFold (Abramson et al., 2024; Jumper et al., 2021) and RoseTTAFold (Krishna et al., 2024) has further expanded the application of AI in this field, enabling accurate prediction of three-dimensional peptide structures that are critical for function. Collectively, these AI driven innovations are reshaping the landscape of drug delivery, opening new avenues for the intelligent design of peptide therapeutics, and enabling more effective and targeted treatments for a variety of diseases.

In this study, we guided LLMs to propose rules for CPPs and derived an integrated ML framework that combines LLM-derived CPP rules with classical sequence features to improve CPP prediction and candidate design. Through targeted prompt engineering, we extracted 20 interpretable rules related to sequence motifs, physicochemical properties, and structural features from GPT-4o and DeepSeek. These rules were encoded as binary vectors and combined with traditional descriptors such as amino acid frequency and physicochemical property to train ML models, including random forest (RF), XGBoost (XGB), support vector machine (SVM), and logistic regression (LR). Benchmarking on a mature CPP dataset showed that the LLM-derived rules outperformed conventional physicochemical and fingerprint descriptors, matched amino-acid composition, and achieved the best overall performance when integrated with composition features (confirmed by repeated cross-validation). Based on these predictive models, we further generated novel CPP sequences that satisfied most of the LLM inference rules. Structural analysis using AlphaFold 3 showed that the newly generated peptides exhibited structural features comparable to classic CPPs such as TAT (Brooks et al., 2005) and Penetratin (Derossi et al., 1998). These computational findings identify sequence-novel candidate carriers for experimental evaluation in intracellular drug delivery. In summary, our study establishes a reproducible LLM-ML framework for AI-guided CPP design and smart drug-delivery research.

## 2 Methods

### 2.1 Overview of the methods

**FIGURE 1:**
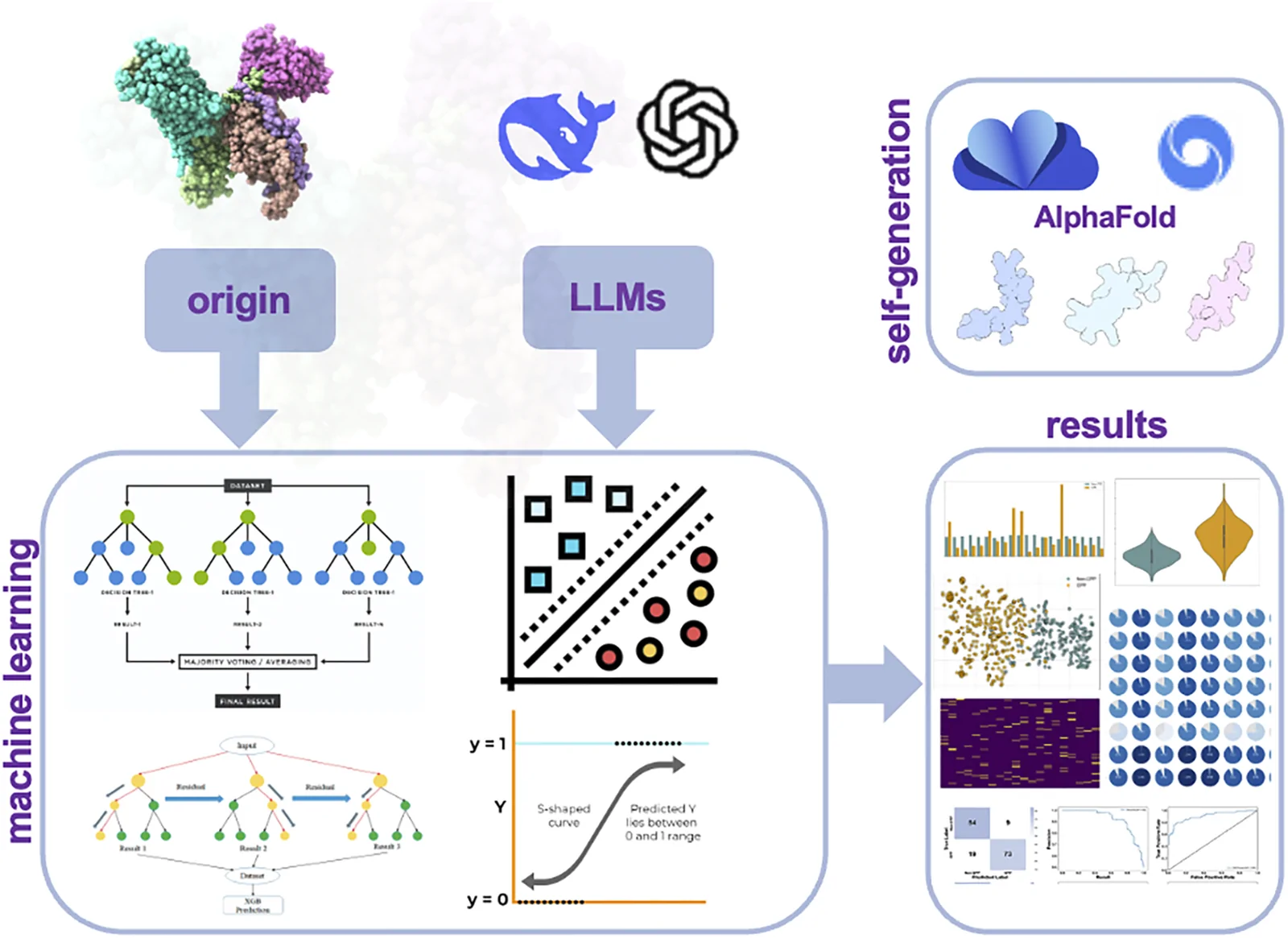
Overview of the study workflow: curation and sequence/physicochemical analysis of CPP924; prompt-engineering of GPT-4o and DeepSeek to derive interpretable CPP rules, encoded as binary vectors; training and cross-validated evaluation of hybrid machine-learning classifiers; and *de novo* generation and computational evaluation of novel CPP candidates.

The main research workflow is illustrated in Figure 1, comprising three major components. Firstly, the dataset used for model training in this study is the stringent benchmark dataset CPP924 (Wei et al., 2017), which is an experimentally validated dataset. We performed property analysis and visualization for both CPP and non-CPP sequences. The second part forms the core of our study, focusing on interactions with GPT-4o and DeepSeek models, along with a comprehensive analysis of the data generated from these interactions. A key aspect of this stage is the art of prompt engineering, involving the design and refinement of prompts to effectively guide GPT-4o (Achiam et al., 2023) and DeepSeek (Liu A. et al., 2024) in formulating accurate and efficient rules for CPP identification. These rules are then vectorized into ML-compatible formats for downstream training and comparative analysis with other input features. The final step involves the generation and analysis of novel CPPs based on the models and rules established.

### 2.2 Data

#### 2.2.1 Dataset

A strictly reliable dataset is crucial for constructing and evaluating prediction models. This study utilizes the widely adopted CPP924 dataset for our experiments. The dataset consists of 924 peptide sequences, equally split into 462 CPP sequences and 462 non-CPP sequences. All data in CPP924 is derived from the largest CPP dataset: CPPsite 2.0 (Agrawal et al., 2016). Importantly, sequence similarity between any two peptides in the dataset is maintained below 80%. This filtering is crucial, as excessive sequence similarity could compromise the model’s predictive performance.

For model development, CPP924 was partitioned into stratified training and test sets (80%/20%; 739/185 sequences; random_state = 42) preserving the 1:1 class balance, and model selection and robustness were assessed by repeated stratified 10-fold cross-validation (5 repeats) on the data.

#### 2.2.2 Sequence and property analysis of CPP and non-CPP

To gain deeper insights into CPPs and their distinguishing features, we conducted a comparative analysis of sequence length, amino acid composition, k-mer distribution, and physicochemical properties across the dataset. Sequence length informs whether peptide length correlates with cell-penetrating ability, influencing both stability and membrane interactions. Amino acid frequency reveals compositional biases, highlighting residues such as arginine and tryptophan, which are commonly enriched in CPPs due to their capacity to interact with lipid bilayers. K-mer analysis of short subsequences enables the identification of functional motifs that may be obscured in full-length sequences. Physicochemical properties, including charge, hydrophobicity, and secondary structure propensity, are fundamental for understanding peptide–membrane interactions. Peptide sequences were one-hot encoded into 20-dimensional binary vectors, zero-padded to uniform length, grouped by class (CPP versus Non-CPP), and visualized as heatmaps using a Viridis color scheme (dark purple = 0, yellow = 1) to highlight positional amino acid patterns. This integrative analysis elucidates how sequence composition and physicochemical attributes contribute to membrane penetration efficiency.

### 2.3 Prompt engineering for defining CPP recognition rules

#### 2.3.1 Zero-shot prompt

To standardize and streamline the conversation process, while also enabling seamless transitions to other models or biochemical tasks in the future, we adopted a standardized zero-shot prompt template: You are an experienced biochemist and peptide delivery expert. Please list 20 rules that you think are most important for predicting the activity of CPPs. Each rule can be about the sequence characteristics, physicochemical properties, or structural characteristics of the peptide. GPT-4o and DeepSeek are positioned as a biochemistry and peptide delivery expert, tasked with developing CPPs recognition rules based on their specialized knowledge in the field.

Reproducibility of rule extraction. The rules were obtained through the standard GPT-4o and DeepSeek interfaces with default decoding settings during the study period; the specific GPT-4o snapshot has since been deprecated by the provider, so re-querying the identical model is no longer possible. A single model-agnostic zero-shot prompt was used deliberately to keep the elicitation transparent, to avoid biasing the models with leading or example-laden prompts, and to permit an identical query across both models. Critically, the conversion of each rule into a binary feature is fully deterministic: all 20 GPT-4o and 20 DeepSeek rules are implemented as explicit Python functions (e.g., “net charge ≥ +4”, “contains an R-x(1,2)-R motif”), provided in the repository, so the rule-to-feature step is unambiguous and reproducible. The two independently-prompted models are concordant (the per-peptide counts of satisfied rules correlate at Pearson r = 0.62), and the rules recapitulate well-established CPP determinants reported in the literature (Milletti, 2012; Guidotti et al., 2017; Copolovici et al., 2014; Bannunah et al., 2014). All prompt logs and rule outputs are provided (see Data and Code Availability).

#### 2.3.2 Conversion of the rule into the 0-1 encoded vector

Transforming CPPs recognition rules into a 0-1 encoding format is crucial for their effective application in ML models. According to the text rules output by GPT -4o and DeepSeek, compliance with the rules is recorded as 1, and non-compliance is recorded as 0. By encoding rules as binary vectors, we convert qualitative and text-based rules into a structured and quantitative format that ML models can directly process.

### 2.4 Machine learning classification models

#### 2.4.1 Processing and analysis of the different inputs

To identify CPPs based on different input features, we developed a binary classification framework. The model incorporated both the rules generated by GPT-4o and DeepSeek as well as a variety of peptide sequence features, including amino acid frequency, amino acid physicochemical property, k-mer pattern, and molecular fingerprint generated using RDKit (Landrum, 2013). These diverse inputs provided the ML algorithms with both LLM derived rules and multi-dimensional representations of the peptide sequences.

In addition, different types of dimensionality reduction are performed on different inputs to observe the separation trend of the labels to determine whether there are significant differences between the different input data themselves in the original high dimensional space. We employed three distinct dimensionality reduction (Van Der Maaten et al., 2009) techniques—Principal Component Analysis (PCA) (Abdi and Williams, 2010), t-Distributed Stochastic Neighbor Embedding (t-SNE) (Van der Maaten and Hinton, 2008) and Uniform Manifold Approximation and Projection (UMAP) (McInnes et al., 2018) on the four different input representations. By projecting the data into two dimensional spaces, we visually examined the clustering patterns and separation trends of the labeled samples. Through dimensionality reduction, it is determined whether each input original high dimensional data itself has a significant distinguishing structure related to the label.

#### 2.4.2 Machine learning models and metrics

For model training, we employed four ML algorithms: Random Forest (RF) (Breiman, 2001), XGBoost (XGB) (Chen and Guestrin, 2016), Support Vector Machine (SVM) (Hearst et al., 1998), and Logistic Regression (LR) (Hosmer et al., 2013). These algorithms were selected for their complementary strengths in handling complex datasets and varying feature spaces. By combining eight types of molecular fingerprints with the four algorithms, a total of 32 model configurations were constructed.

Model training and selection used a stratified 80/20 train/test split (739/185; random_state = 42) together with repeated stratified 10-fold cross-validation (5 repeats; 50 folds; seed 42); we report mean ± standard deviation for all metrics and use paired t-tests and Wilcoxon signed-rank tests to compare feature sets. Hyper-parameters were fixed and are listed in the SI (e.g., Random Forest: n_estimators = 150, max_depth = 10, class_weight = ‘balanced’, out-of-bag scoring enabled). To isolate the contribution of the LLM-derived rules, we additionally evaluated (i) a continuous physicochemical-descriptor baseline and (ii) a shuffled-rules control in which the rule vectors were randomly permuted across peptides.

Each trained model was evaluated on an independent test set. Evaluation metrics included accuracy (Streiner and Norman, 2006), balanced accuracy (Brodersen et al., 2010), precision (Streiner and Norman, 2006; Buckland and Gey, 1994; Zhu, 2004), recall (Buckland and Gey, 1994; Zhu, 2004), F1 score (Chicco and Jurman, 2020), Matthews Correlation Coefficient (MCC) (Chicco and Jurman, 2020), area under the ROC curve (AUC) (Bradley, 1997), and average precision (Zhu, 2004). To further illustrate model performance, we plotted Receiver Operating Characteristic (ROC) curves (Fan et al., 2006), Precision-Recall (Buckland and Gey, 1994) (PR) curves and confusion matrices (Townsend, 1971). This comprehensive evaluation enabled us to systematically compare the predictive power of different input features and to assess the effectiveness of each ML model in distinguishing CPP from non-CPP. Ultimately, it allowed us to identify the optimal combination of fingerprint type and classification algorithm for CPPs prediction.

### 2.5 Generation and evaluation of novel CPP based on the model and rules

The best performing models, RF-GPT-Fre and RF-DS-Fre, were employed to generate new CPPs based on the corresponding generation rules. The structures of the newly designed peptides were subsequently predicted and analyzed using AlphaFold 3 (Abramson et al., 2024) to assess their conformational characteristics and potential functionality. The generated structures were visualized using the software ChimeraX (Pettersen et al., 2021). To comprehensively evaluate the potential of the generated peptides as CPPs, we utilized two widely recognized prediction platforms, CellPPD (Gautam et al., 2013; Gautam et al., 2015) and C2Pred (Tang et al., 2016), with all parameters set to their default values. Furthermore, peptide toxicity and allergenicity were assessed using established online analysis tools: ToxinPred (Gupta et al., 2013) and AllerTOP (Dimitrov et al., 2014).

## 3 Results and discussion

### 3.1 Sequence and property analysis of CPP and non-CPP

**FIGURE 2:**
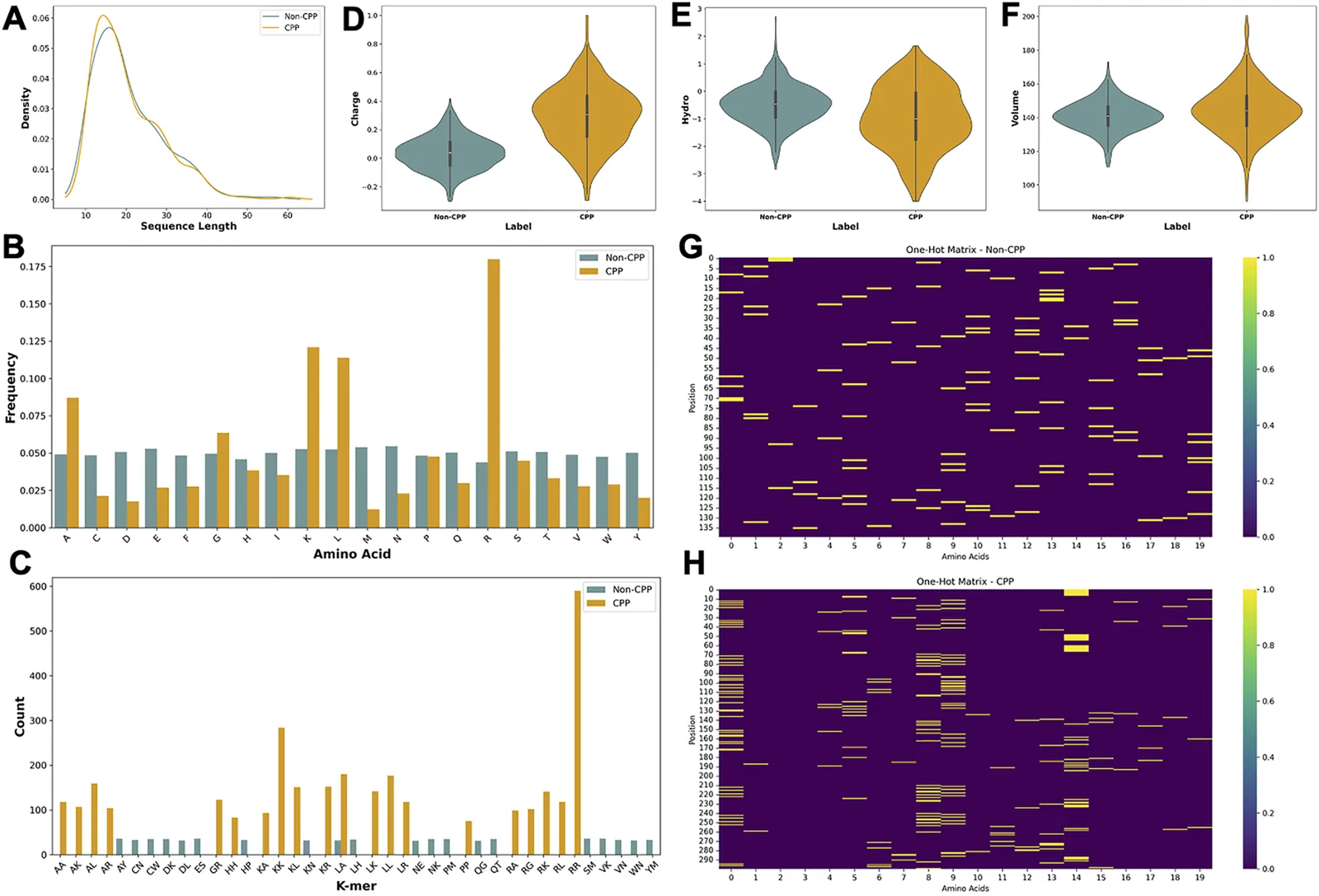
Sequence and physicochemical analysis of CPP (orange) versus non-CPP (green) sequences. **(A)** Sequence-length distribution; **(B)** amino-acid frequency; **(C)** k-mer (k = 2) distribution; **(D)** net charge; **(E)** hydrophobicity; **(F)** peptide volume; **(G,H)** one-hot positional amino-acid heatmaps for non-CPP **(G)** and CPP **(H)** sequences (Viridis scale: dark = 0, yellow = 1).

The sequence and physicochemical properties of CPPs and non-CPPs were systematically analyzed. As shown in Figure 2A, peptide sequence length distributions are generally similar between the two classes, with CPPs exhibiting slightly shorter sequences on average. Figure 2B shows that CPPs (orange) are enriched in positively charged residues, such as arginine (R) and lysine (K), at significantly higher frequencies than non-CPPs (green). This enrichment likely enhances interactions with negatively charged cell membranes, facilitating cellular uptake. In contrast, non-CPPs display higher proportions of hydrophobic residues, including valine (V) and isoleucine (I), which may hinder membrane penetration. These findings highlight the presence of positively charged amino acids as a key characteristic of CPP, supporting the hypothesis that electrostatic interactions drive cellular internalization (Bannunah et al., 2014).

K-mer analysis (K = 2; Figure 2C) revealed that CPPs contain higher counts of specific motifs, such as consecutive arginine residues, suggesting that short sequence patterns are critical for penetration efficiency. Non-CPPs exhibited a more dispersed distribution, lacking prominent motifs. Physicochemical analysis further corroborated these findings. Net charge analysis (Figure 2D) shows that CPPs carry significantly positive charges, consistent with amino acid composition, whereas non-CPPs are near-neutral or slightly negative. Similarly, non-CPPs exhibit higher hydrophobicity (Figure 2E), limiting membrane penetration, while peptide volume (Figure 2F) appears to exert minimal influence.

One-hot encoding matrices revealed distinct positional preferences: CPP sequences show clustered high-frequency residues at specific positions (Figure 2H), whereas non-CPPs display sparse, scattered distributions (Figure 2G). This contrast indicates that CPPs possess localized amino acid patterns, while non-CPPs exhibit more uniform but less dense distributions, reflecting potential structural or functional differences. Collectively, these analyses of sequence and physicochemical features provide critical insights into the determinants of CPP function and inform the rational design of peptide-based delivery systems.

### 3.2 Prompt engineering for defining CPP recognition rules

**FIGURE 3:**
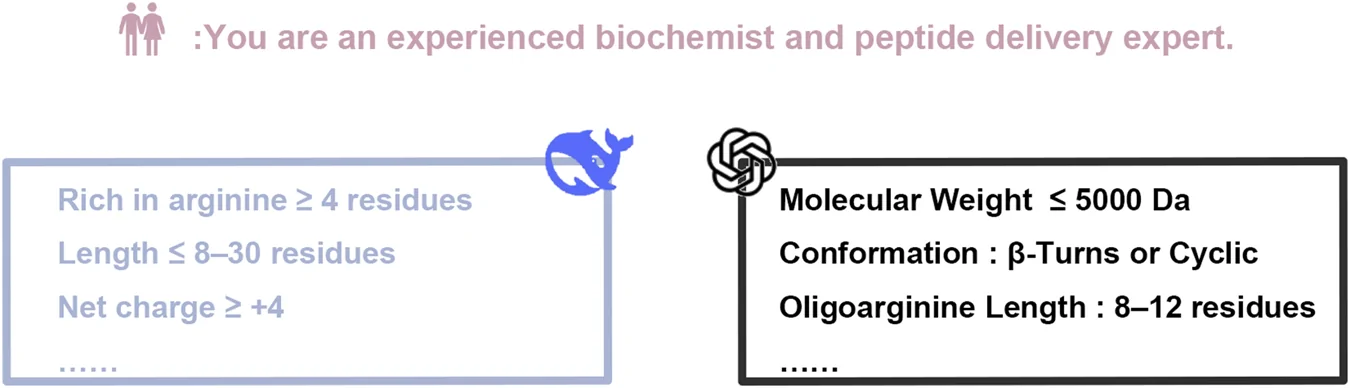
Prompt-engineering scheme used to elicit 20 interpretable CPP rules from GPT-4o and DeepSeek, each acting as a peptide-delivery expert under an identical zero-shot prompt.

Using prompt engineering, we guided two large language models (LLMs) to generate CPP-specific rules. Each model produced 20 rules encompassing sequence characteristics, physicochemical properties, and structural features of peptides. As illustrated in Figure 3, GPT-4o and DeepSeek were systematically engaged via prompt engineering to derive these interpretable CPP-related rules.

### 3.3 Comparison of outputs based on the models and peptide types

**FIGURE 4:**
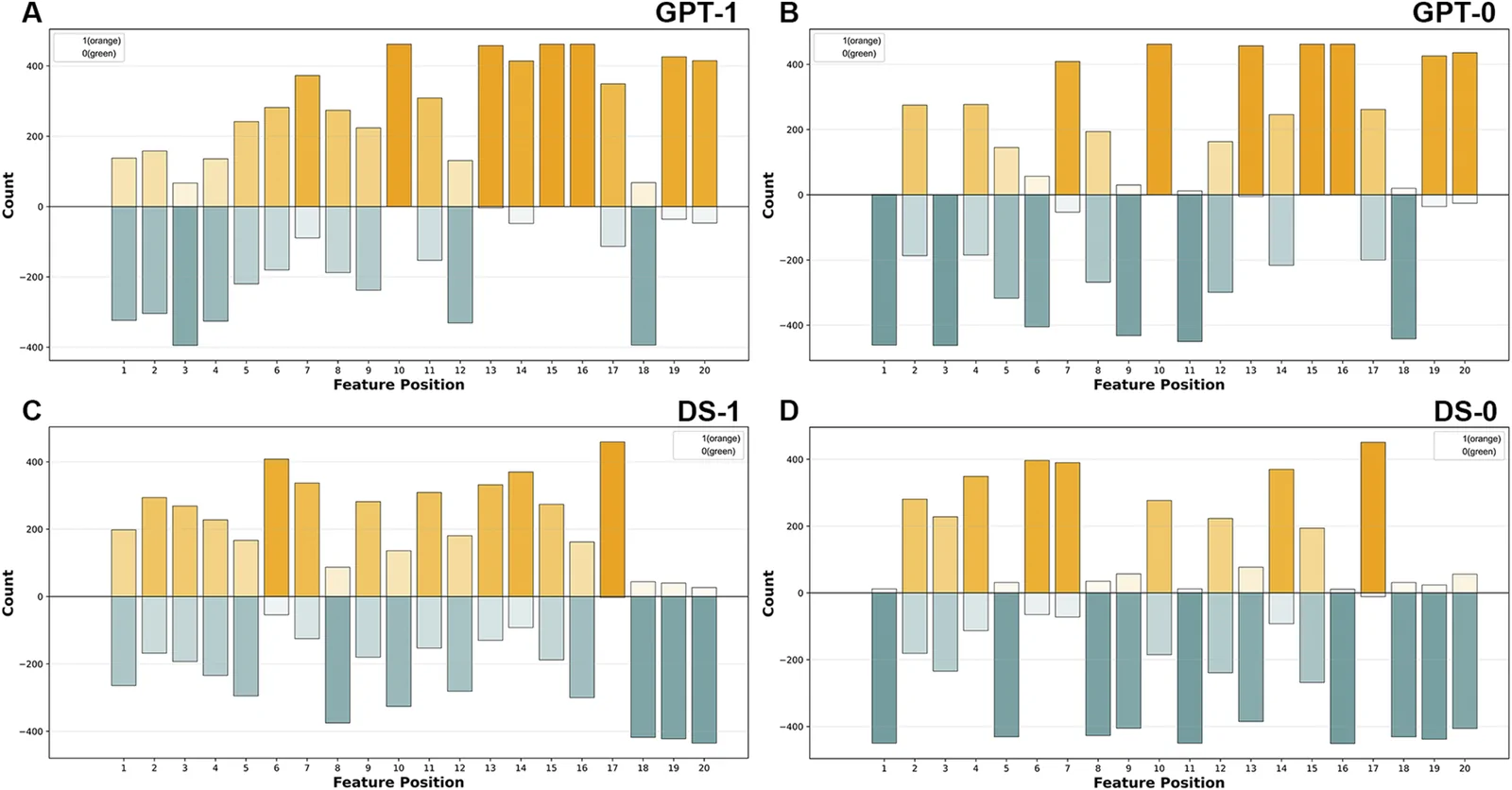
Binary-encoded compliance of CPP and non-CPP sequences with the LLM-derived rules. **(A,B)** GPT-4o rules for CPP **(A)** and non-CPP **(B)** sequences; **(C,D)** DeepSeek rules for CPP **(C)** and non-CPP **(D)** sequences. Detailed rule descriptions are given in Supplementary Table S1.

The 20 rules generated by the two models were applied to both CPP and non-CPP sequences and subsequently converted into binary encoding. Twenty-dimensional binary encoding was visualized to compare CPP and non-CPP distributions under each rule. As shown in Figures 4A,B, CPP sequences more frequently complied with Rules 1, 3, 9, and 14, while non-CPP sequences more frequently complied with Rules 1, 3, 6, 9, and 11. As shown in Figures 4C,D, CPP sequences more frequently complied with Rules 1, 5, 9, 11, 13 and 14, while non-CPP sequences more frequently complied with Rules 1, 5, 9, 11, 13 and 16. More detailed rules are shown in Supplementary Table S1. Although some rules exhibited only minor, descriptive distributional differences between CPP and non-CPP sequences (no formal enrichment test was applied; see Limitations), we retained all features to assess the overall utility of LLM-derived rules in ML applications.

### 3.4 Dimensionality reduction on different inputs

**FIGURE 5:**
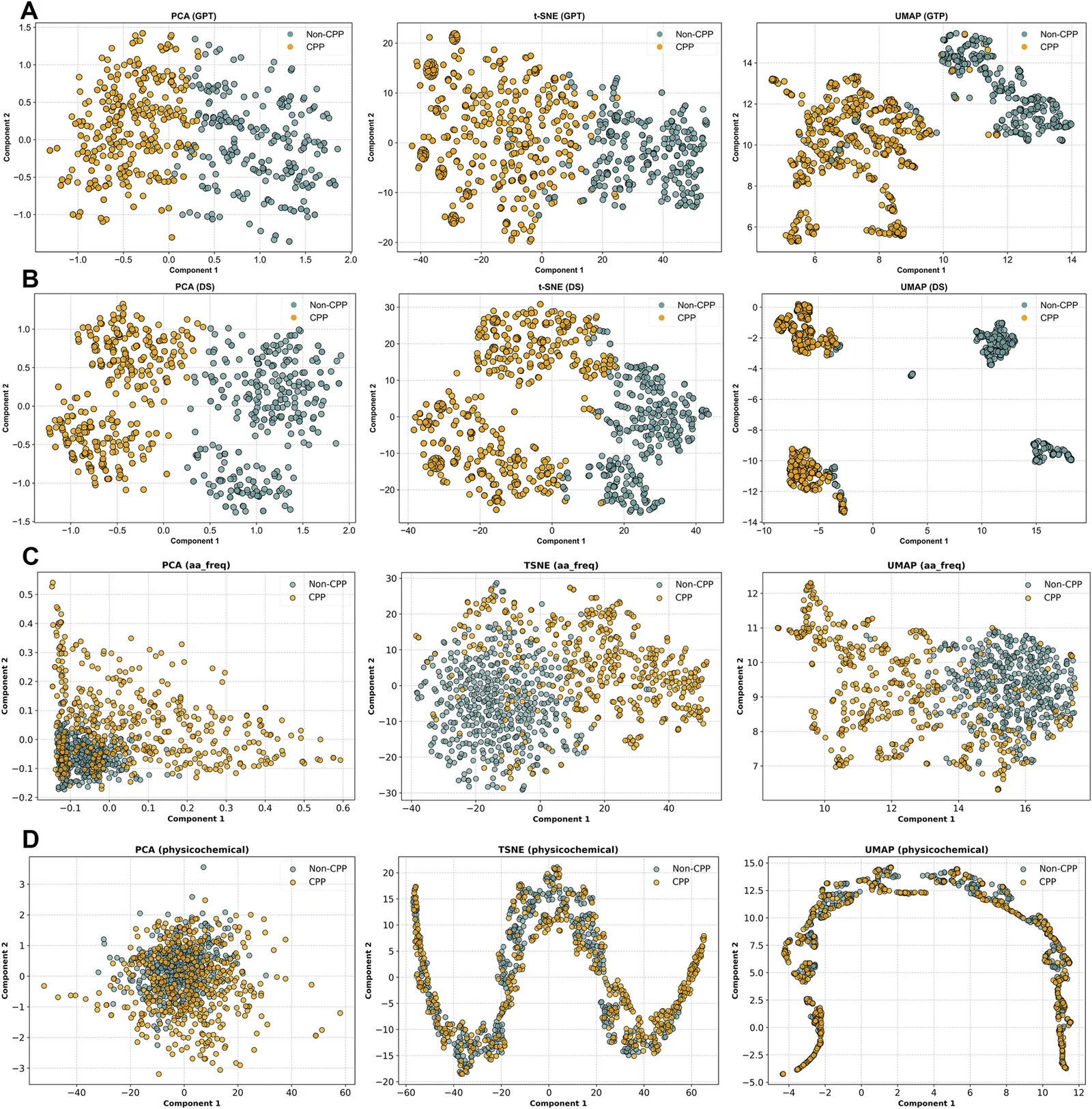
Two-dimensional projections (PCA, t-SNE, UMAP) of the input encodings, coloured by class: **(A)** GPT-4o rules; **(B)** DeepSeek rules; **(C)** amino-acid frequency; **(D)** physicochemical properties.

To further evaluate the effectiveness of the rules generated by the LLMs in distinguishing between CPP and non-CPP sequences, we applied three different dimensionality reductions. As shown in Figures 5A,B, the rules generated by GPT-4o and DeepSeek effectively capture the distinction between CPP and non-CPP sequences, indicating that the encoding in high-dimensional space is closely aligned with labels. Additionally, we applied dimensionality reductions to the data encoded based on amino acid frequency and physicochemical properties, and compared these results with those derived from regular encoding. For amino acid frequency (Figure 5C), t-SNE is able to partially separate the labels, while PCA and UMAP yield suboptimal results. In comparison, for physicochemical properties Figure 5D, none of the three dimensionality reduction methods provide satisfactory separation. Based on the results, the rules generated by GPT-4o and DeepSeek effectively distinguish between CPP and non-CPP sequences, with the encoding aligning well with the labels in high dimensional space.

### 3.5 Performance evaluation of classification models

**FIGURE 6:**
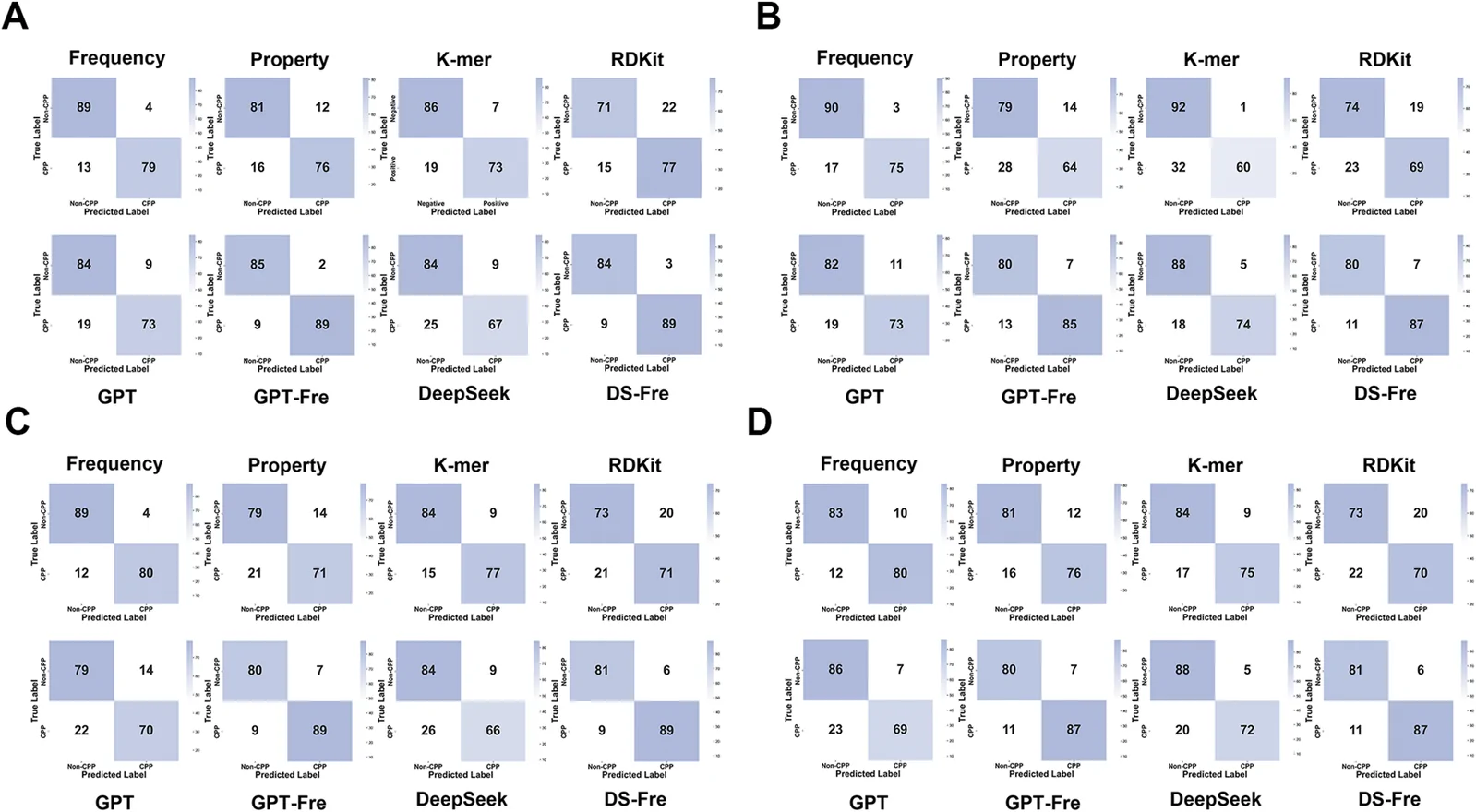
Confusion matrices for the four classifiers - **(A)** RF, **(B)** SVM, **(C)** XGB and **(D)** LR. Within each panel, the eight matrices correspond to four sequence descriptors (amino-acid frequency, physicochemical property, k-mer, RDKit) and the four rule/integrated encodings (GPT, GPT-Fre, DeepSeek, DS-Fre).

Combining the four sequence feature inputs with different ML models, we found that the amino acid frequency representation achieved the best overall performance. When using the rule-based encoders generated by GPT and DeepSeek as input, the results were comparable to those of amino acid frequency. In some ML models, the rule-based encoders even outperformed amino acid frequency. Therefore, we selected a combined input of rule encoding and amino acid frequency for subsequent ML model training to maximize predictive performance. As shown in Figure 6, for the RF model, the amino acid frequency input performed comparably to the rule-based encoder generated by GPT-4o and DeepSeek. When the two feature sets were combined, the models using GPT-Fre and DeepSeek-Fre outperformed those using amino acid frequency alone, particularly in the classification of CPP. For the SVM model, the amino acid frequency input performed comparably to the rule-based encoders generated by GPT-4o and DeepSeek, with the rules output by DeepSeek showing particularly strong performance. When the two feature sets were combined, the classification of CPP sequences was improved. However, the accuracy for non-CPP sequences slightly declined. Similar to the SVM model, in the XGB model, the amino acid frequency input outperformed the rule-based encoders generated by GPT-4o and DeepSeek. However, when the two feature sets were combined, the classification of CPP sequences was significantly improved, while the classification performance for non-CPP sequences declined. In the LR model, the amino acid frequency input outperformed the rule-based encoders generated by GPT-4o and DeepSeek, particularly in the classification of CPP sequences. When the two feature sets were combined, the classification of CPP sequences was significantly improved, while the performance for non-CPP sequences decreased. In more detail, Supplementary Figure S1 presents the ROC curves and precision-recall curves for various model and input combinations.

The classification performance of the four ML models, when using a combination of rule encoding and amino acid frequency, is superior to using either feature set alone, particularly in the discrimination of CPP samples. Overall, based on the confusion matrix, the RF model demonstrates the best performance when both rule encoding and amino acid frequency are used as input: RF-GPT-Fre and RF-DS-Fre.

**FIGURE 7:**
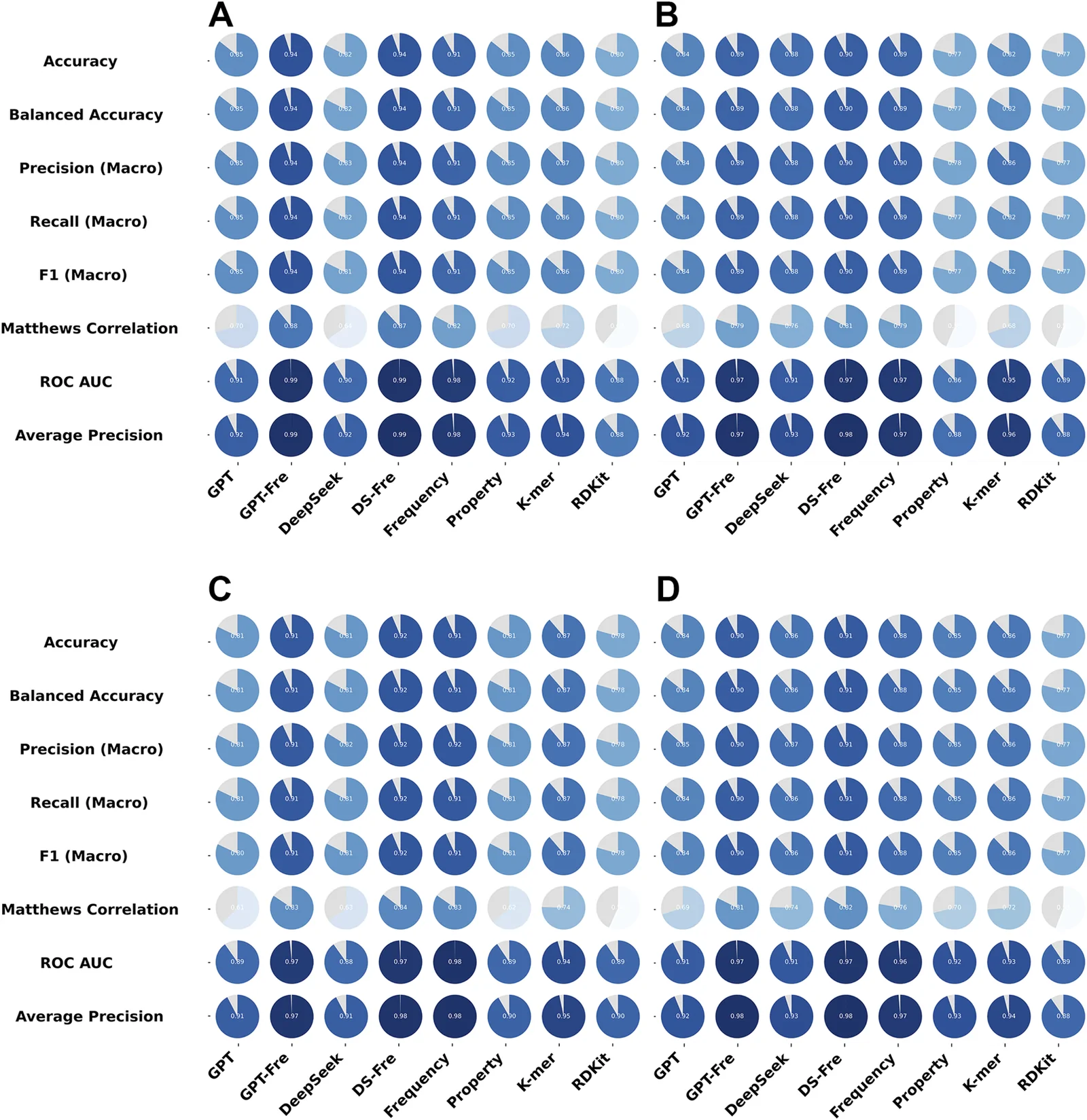
Cross-validated performance metrics (mean ± SD; accuracy, balanced accuracy, F1, AUC, MCC) for all classifier × feature-set combinations; RF-GPT-Fre and RF-DS-Fre give the best overall performance. **(A)** RF, **(B)** SVM, **(C)** XGB and **(D)** LR.

Figure 7 provides a detailed overview of machine learning evaluation metrics and shows that RF-GPT-Fre and RF-DS-Fre deliver the best overall performance. On the held-out test split the two models reach an accuracy of 0.94; under repeated stratified cross-validation they achieve 0.912 ± 0.033 (RF-GPT-Fre) and 0.913 ± 0.029 (RF-DS-Fre) with AUC ≈ 0.97 (Table 1). Among the other three models, DS-Fre performs best in SVM, XGB, and LR, with accuracies of 0.90, 0.92, and 0.91, respectively. These results show that the DeepSeek-derived rules and amino-acid-frequency features provide a robust integrated representation across model classes. Together, RF-GPT-Fre and RF-DS-Fre constitute the selected classifiers for subsequent candidate generation and evaluation.

**TABLE 1:** Cross-validated accuracy (mean ± SD; repeated stratified 10-fold × 5) for each classifier and feature set.

Feature set	RF	SVM	XGB	LR
Phys	0.822 ± 0.038	0.827 ± 0.034	0.818 ± 0.037	0.809 ± 0.034
k-mer	0.860 ± 0.034	0.869 ± 0.028	0.852 ± 0.031	0.841 ± 0.034
Fre	0.906 ± 0.028	0.912 ± 0.025	0.915 ± 0.028	0.893 ± 0.030
GPT	0.846 ± 0.038	0.844 ± 0.036	0.830 ± 0.036	0.840 ± 0.033
DS	0.860 ± 0.035	0.848 ± 0.034	0.850 ± 0.033	0.856 ± 0.029
GPT-Fre	0.912 ± 0.033	0.906 ± 0.027	0.919 ± 0.031	0.896 ± 0.029
DS-Fre	0.913 ± 0.029	0.909 ± 0.027	0.916 ± 0.029	0.900 ± 0.029

AUC and MCC are given in Supplementary Table S3.

To quantify the contribution of the LLM-derived rules, we performed controlled feature analyses (Table 1). The rule encodings outperformed a continuous physicochemical-descriptor baseline (0.846 vs. 0.822 for GPT rules, paired Wilcoxon p < 0.01; 0.860 vs. 0.822 for DeepSeek rules, p < 1 × 10^−5^) and matched amino-acid composition. The shuffled-rules control, in which rule vectors were permuted across peptides, confirmed rule-specific contribution for SVM and logistic regression and indicated partial redundancy with sequence composition for tree-based models. Feature-importance analysis identified the LLM rule “net charge ≥ +4” as the most informative feature (importance 0.17), with the rule block contributing ∼36% of total importance. The LLM-derived rules are therefore interpreted as an interpretable, biochemically grounded feature set and a generative design prior. On the same CPP924 benchmark, our cross-validated accuracy (0.91) is competitive with previously reported CPP predictors (CPPred-RF (Wei et al., 2017), DeepCPPred (Arif et al., 2021), EnDM-CPP (Zhu et al., 2024), GraphCPP (Imre et al., 2025)); a literature-based comparison is provided in place of re-implementing external tools under different protocols. As a direct baseline requested during review, we additionally asked two current-version LLMs to classify the held-out test peptides from sequence alone: GPT-4o and DeepSeek-V3 reached accuracies of 0.854 and 0.773, respectively, both below the integrated ML models, with high precision but low recall (Supplementary Table S10). This result supports operationalising LLM-derived rules within a deterministic classical classifier rather than querying an LLM directly.

### 3.6 The generation and evaluation of novel CPPs

**FIGURE 8:**
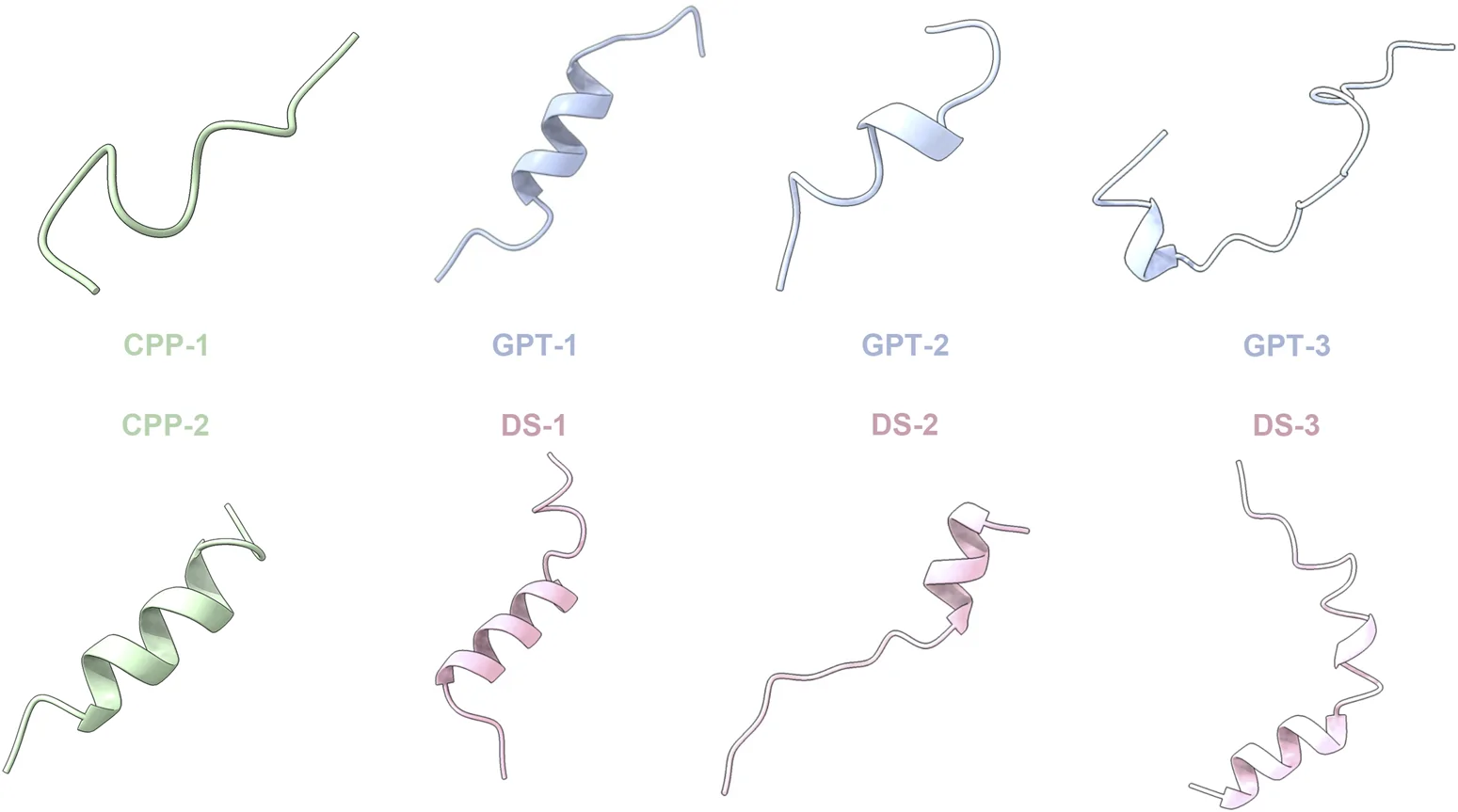
AlphaFold-3 structural models (Model 0) of the six *de novo* peptides (GPT-1–3, DS-1–3) and the reference CPPs TAT (CPP-1) and Penetratin (CPP-2), visualised in ChimeraX. Mean per-residue pLDDT and TM-align similarity metrics are reported in Table 3; GPT-1, DS-1 and DS-3 align most closely with Penetratin.

Finally, candidate peptides were generated by a reproducible rejection-sampling procedure: sequences were drawn with length sampled from the empirical CPP length distribution (10–61 aa) and residues from the CPP-class composition prior, scored against the 20 operationalised rules, and retained only if they satisfied at least 15 of the 20 rules; the retained candidates were then ranked by the two best-performing models (RF-GPT-Fre and RF-DS-Fre), de-duplicated and screened for novelty. The rule-filter acceptance rate is 30.5% (28.0% additionally pass a classifier-probability threshold of 0.80), and 2.05% under a uniform-residue prior, quantifying the constraint imposed by the rules. The selected sequences and their predicted probabilities are listed in Table 2, where “Probability” denotes the classifier’s posterior probability of the CPP class; each candidate satisfies 15–18 of the 20 rules. Sequence novelty was quantified by global-alignment identity against all 924 training peptides: the maximum identity is 35.7%–53.3% (≤30% to TAT, ≤ 19% to Penetratin), establishing that the candidates are new sequences. AlphaFold-3 structural models (Model 0) were obtained with high per-residue confidence (mean pLDDT 82.3–95.3) and visualised with ChimeraX (Figure 8); two classical CPPs served as references, CPP-1 (TAT (Brooks et al., 2005), “YGRKKRRQRRR”) and CPP-2 (Penetratin (Derossi et al., 1998), “RQIKIWFQNRRMKWKK”). Quantitative structural comparison by TM-align (Table 3) shows that GPT-1 and DS-1 are more similar to Penetratin than to TAT (TM-score 0.46 and 0.49 versus 0.25 and 0.24; Cα-RMSD 1.64 Å and 0.80 Å), and DS-3 likewise aligns closely with Penetratin (TM-score 0.45; Cα-RMSD 0.63 Å), consistent with their composition- and rule-level similarity to classical cationic CPPs. These results establish a computational candidate-prioritisation workflow for subsequent experimental validation.

**TABLE 2:** The peptide sequences and scores predicted by the best-performing model.

Model	ID	Sequence	Probability
GPT-Fre	GPT-1	TRRRLPIILAHNRRRPFH	0.87
	GPT-2	RRRRSHKNRPCR	0.86
	GPT-3	SFFRHKRRRLTTHWPRNLSRRVI	0.86
DS-Fre	DS-1	MLPATRLQTARRRRKAPRQRAQ	0.83
	DS-2	FPHAPRRRGPRISKRIFR	0.79
	DS-3	GGRKGRWRLVTPLILPRRRIAQHINWT	0.78

“Probability” is the classifier-predicted posterior probability of the CPP class.

**TABLE 3:** AlphaFold-3 (Model 0) confidence and structural-similarity metrics.

ID	Mean pLDDT	TM-score vs. TAT	TM-score vs. penetratin	Cα-RMSD vs. penetratin (Å)
GPT-1	88.0 ± 2.5	0.25	0.46	1.64
GPT-2	82.3 ± 5.0	0.27	0.21	1.31
GPT-3	85.9 ± 3.4	0.20	0.28	1.10
DS-1	95.3 ± 2.1	0.24	0.49	0.80
DS-2	91.8 ± 2.6	0.35	0.32	1.74
DS-3	86.3 ± 5.9	0.26	0.45	0.63
CPP-1 (TAT)	89.0 ± 2.9	—	—	—
CPP-2 (penetratin)	94.9 ± 3.0	—	—	—

pLDDT is the mean ± SD per-residue value; TM-score and Cα-RMSD are from TM-align against the reference CPPs.

To evaluate the generated peptides with established computational predictors, we used CellPPD (Gautam et al., 2013; Gautam et al., 2015), C2Pred (Tang et al., 2016), ToxinPred (Gupta et al., 2013), and AllerTOP (Dimitrov et al., 2014). As presented in Table 4, CellPPD classified the generated peptides as CPPs with scores of 0.11–0.68, and C2Pred classified all six generated peptides as CPPs with scores of 0.68–0.99. ToxinPred classified the generated peptides as non-toxic, and AllerTOP classified all generated peptides as non-allergenic except DS-3, which was identified as a potential allergen. These *in silico* evaluations provide computational prioritisation evidence; functional CPP activity will be evaluated by cellular uptake, cytotoxicity, and cargo-delivery assays.

**TABLE 4:** The predicted type and score from other CPP design models, along with peptide toxicity and allergenicity analyses obtained from peptide evaluation platforms.

ID	Sequence	CellPPD type	CellPPD score	C2Pred type	C2Pred score	ToxinPred type	ToxinPred score	AllerTOP type
GPT-1	TRRRLPIILAHNRRRPFH	CPP	0.11	CPP	0.98	Non-toxin	−0.64	Non-allergen
GPT-2	RRRRSHKNRPCR	CPP	0.68	CPP	0.68	Non-toxin	−0.84	Non-allergen
GPT-3	SFFRHKRRRLTTHWPRNLSRRVI	CPP	0.13	CPP	0.93	Non-toxin	−1.14	Non-allergen
DS-1	MLPATRLQTARRRRKAPRQRAQ	CPP	0.25	CPP	0.99	Non-toxin	−1.21	Non-allergen
DS-2	FPHAPRRRGPRISKRIFR	CPP	0.22	CPP	0.90	Non-toxin	−2.06	Non-allergen
DS-3	GGRKGRWRLVTPLILPRRRIAQHINWT	CPP	0.24	CPP	0.74	Non-toxin	−1.43	Allergen
CPP-1	YGRKKRRQRRR	CPP	1.07	CPP	0.98	Non-toxin	−0.65	Non-allergen
CPP-2	RQIKIWFQNRRMKWKK	CPP	1.32	CPP	0.91	Non-toxin	−0.66	Non-allergen
Non-CPP-1	FGIHSPVNWLANARF	Non-CPP	−0.32	Non-CPP	0.81	Non-toxin	−0.92	Non-allergen
Non-CPP-2	MRVCHIYQADYKDGDAHDH	Non-CPP	−0.51	Non-CPP	0.88	Non-toxin	−0.97	Allergen

## 4 Conclusion

In this study, we investigated the properties and sequences of CPPs and non-CPPs and developed an AI framework that synergizes LLMs with ML techniques for CPP prediction and rational design. Through targeted prompt engineering, 20 biochemical rules governing CPP functionality were extracted from GPT-4o and DeepSeek, systematically encoded, and combined with classical sequence-derived features to train high-performance classifiers. Our best models (RF-GPT-Fre and RF-DS-Fre) achieved a cross-validated accuracy of 0.91 ± 0.03 (best held-out split, 0.94) on the CPP924 dataset; the LLM-derived rules outperformed conventional physicochemical and fingerprint descriptors, matched amino-acid composition, and delivered the best overall performance when integrated with composition features. Beyond predictive modeling, we generated six sequence-novel CPP candidates (≤53% identity to any training peptide) that satisfied the majority of the LLM-derived rules. Structural prediction and computational characterisation established their resemblance to known CPPs in composition and structure, and these candidates are prioritised for experimental validation of cellular uptake.

This study demonstrates the use of artificial intelligence to accelerate peptide discovery and to decipher sequence–function relationships. By integrating interpretable LLM-derived design rules with classical sequence descriptors, the framework provides a scalable and mechanistically transparent strategy for prioritising candidate functional peptides. The framework is extensible to antimicrobial, cell-targeting and cancer-selective peptide design. The present results are computational, and the generated candidates are prioritised for experimental testing, including fluorescence-based cellular-uptake, cytotoxicity and cargo-delivery assays. The framework provides an interpretable computational foundation to guide the design of cell-penetrating peptides for drug delivery.

### 4.1 Limitations

This work has the following scope and limitations. (1) The study is computational; experimental cell-uptake, cytotoxicity and cargo-delivery assays constitute the functional validation step. (2) Model development uses the CPP924 benchmark, in which all peptide pairs share <80% identity; as CPPsite 2.0/CPP924 are public, the rules may overlap with the LLMs’ pre-training corpora, and this is stated explicitly. (3) The LLM-derived rules encode determinants that overlap with sequence composition (the number of satisfied rules correlates with arginine content and net charge, r ≈ 0.6–0.7); their value lies in interpretability and in serving as a generative design prior, in addition to classification. (4) Downstream evaluation of the generated peptides uses computational predictors (CellPPD, C2Pred, ToxinPred, AllerTOP); experimental assays provide functional validation. (5) AlphaFold-3 structural similarity characterises conformational resemblance and is interpreted together with the sequence- and composition-level evidence. (6) The GPT-4o snapshot used is deprecated; the rules are therefore provided as deterministic code, and robustness is supported by cross-model concordance, with protein-language-model comparisons identified as future work. We also benchmarked direct LLM classification: when current-version GPT-4o and DeepSeek-V3 are asked to label the test sequences on their own, they are less accurate than—and, being version-dependent, less reproducible than—the rule-based ML pipeline (Supplementary Table S10), reinforcing the value of operationalising the rules as deterministic code.

### 4.2 Data and Code Availability

The CPP924 benchmark is publicly available from CPPsite 2.0. All code and data required to reproduce this study–dataset splits, preprocessing and feature-encoding scripts, the verbatim LLM prompt templates and raw rule outputs, the rule-to-binary conversion logic for all 40 rules, model-training and evaluation scripts (with fixed seeds and hyper-parameters), the *de novo* generation code, and the scripts reproducing the cross-validation, shuffled-rules control, feature-importance, novelty and learning-curve analyses–are available at https://github.com/hhy507/LLM-CPP-prediction.

## Data Availability

The original contributions presented in the study are included in the article/Supplementary Material, further inquiries can be directed to the corresponding authors.
