# Supplementary material for: Artificial intelligence-driven prediction and design of cell-penetrating peptides for advanced drug delivery system
Source: Front Pharmacol. 2026 Aug 26;17:1845913. doi: 10.3389/fphar.2026.1845913 (PMC13555210; doi:10.3389/fphar.2026.1845913)
Supplement: Supplementary file 1 [file Supplementaryfile1.docx]

**Supplementary Materials**

**Contents**

• Table S1. Complete LLM-derived CPP rules (GPT-4o and DeepSeek) and their operational binary criteria

• Table S2. Classifier hyper-parameters and model-selection rule

• Table S3. Cross-validated AUC and MCC for all classifier × feature-set combinations

• Table S4. Shuffled-rules control

• Table S5. Paired significance tests (t-test and Wilcoxon)

• Table S6. Held-out test-split metrics

• Table S7. Low-data learning curve

• Table S8. Sequence novelty of the de novo candidates

• Table S9. De novo generation acceptance rates

• Figure S1. ROC and precision–recall curves (high resolution)

• Figures S2–S7. Supporting analyses (cross-validation, shuffled control, learning curve, novelty, AlphaFold-3 confidence and structural similarity)

**Table S1. LLM-derived CPP rules and operational criteria**

Each natural-language rule is implemented as a deterministic Python function; compliance is encoded as 1 and non-compliance as 0. This is the corrected Table S1 (cf. Reviewer 5, Comment 2).

**Table S1a. The 20 GPT-4o-derived rules.**

| **#** | **GPT-4o rule (natural language)** | **Operational binary criterion** |
| --- | --- | --- |
| **G1** | Arginine count ≥ 6 | count(R) ≥ 6 |
| **G2** | Contains at least one tryptophan | count(W) > 0 |
| **G3** | Arginine count between 8 and 12 | 8 ≤ count(R) ≤ 12 |
| **G4** | Contains at least one histidine | count(H) > 0 |
| **G5** | More arginines than lysines | count(R) > count(K) |
| **G6** | No acidic residues (Asp/Glu) | count(D)+count(E) = 0 |
| **G7** | Hydrophobic-residue count between 1 and 10 | 1 ≤ count(F,I,L,V,W,Y) ≤ 10 |
| **G8** | Cationic–hydrophobic adjacency motif | regex [RK][FILVWY] present |
| **G9** | Arginine pairing motif R-x(1,2)-R | regex R.{1,2}R present |
| **G10** | Terminal modification assumed (N-acetyl/C-amide) | constant = 1 (design assumption) |
| **G11** | Net charge ≥ +4 | net_charge ≥ 4 |
| **G12** | Contains both Arg and Phe (hydrophobic-moment proxy) | R ∈ seq and F ∈ seq |
| **G13** | Molecular weight < 5000 Da | length × 110 < 5000 |
| **G14** | Predicted isoelectric point pI > 9 | pI(estimate) > 9 |
| **G15** | Balanced mean hydrophobicity | -1 < mean_hydrophobicity < 1 |
| **G16** | Predicted stable (proteolytic-stability assumption) | constant = 1 (design assumption) |
| **G17** | Run of ≥4 helix/amphipathicity-favouring residues | regex [AILMKRHFYVW]{4,} present |
| **G18** | First residue equals last residue (cyclisation proxy) | seq[0] == seq[-1] |
| **G19** | Length < 35 residues | length < 35 |
| **G20** | No GG dipeptide (aggregation avoidance) | 'GG' not in seq |

**Table S1b. The 20 DeepSeek-derived rules.**

| **#** | **DeepSeek rule (natural language)** | **Operational binary criterion** |
| --- | --- | --- |
| **D1** | Arginine count ≥ 4 | count(R) ≥ 4 |
| **D2** | Contains at least one lysine | count(K) > 0 |
| **D3** | Low overall hydrophobicity | hydrophobicity < 0.4 |
| **D4** | Amphipathicity window | 0.3 < hydrophobicity < 0.6 |
| **D5** | No aromatic residues (W/F/Y) | aromatic_count = 0 |
| **D6** | Length between 8 and 30 residues | 8 ≤ length ≤ 30 |
| **D7** | Contains proline or glycine (turn propensity) | count(P) > 0 or count(G) > 0 |
| **D8** | Balanced cationic/hydrophobic ratio | 0.8 ≤ cationic/hydrophobic ≤ 1.2 |
| **D9** | No anionic residues (Asp/Glu) | anionic_count = 0 |
| **D10** | Contains at least one histidine | count(H) > 0 |
| **D11** | Net charge ≥ +4 | net_charge ≥ 4 |
| **D12** | Hydrophobicity between 0.2 and 0.4 | 0.2 ≤ hydrophobicity ≤ 0.4 |
| **D13** | Combined Arg+Lys ≥ 4 (simplified high pI) | count(R)+count(K) ≥ 4 |
| **D14** | Molecular weight < 3000 Da | length × 110 < 3000 |
| **D15** | Cationic–hydrophobic adjacency motif (PSA proxy) | regex [RK][FILVWY] present |
| **D16** | Polycationic run (helix proxy) | regex [RK]{3,} present |
| **D17** | No acidic run (β-sheet avoidance) | regex [ED]{3,} absent |
| **D18** | Proline turn motif P-x(1,2)-P | regex P.{1,2}P present |
| **D19** | Starts with alanine (N-terminal-modification proxy) | seq.startswith('A') |
| **D20** | Cysteine pair for cyclisation C-x(3,7)-C | regex C.{3,7}C present |

**Table S2. Hyper-parameters and model selection**

**Table S2. Fixed hyper-parameters per classifier; selection criterion = highest mean cross-validated balanced accuracy.**

| **Classifier** | **Key hyper-parameters** | **Selection** |
| --- | --- | --- |
| **Random Forest** | n_estimators=150, max_depth=10, class_weight='balanced', OOB enabled | highest mean CV balanced accuracy |
| **XGBoost** | n_estimators=200, max_depth=6, learning_rate=0.1, subsample=0.8 | highest mean CV balanced accuracy |
| **Support Vector Machine** | kernel='rbf', C=1.0, gamma='scale', probability=True | highest mean CV balanced accuracy |
| **Logistic Regression** | penalty='l2', C=1.0, max_iter=1000, class_weight='balanced' | highest mean CV balanced accuracy |

**Table S3. Cross-validated AUC and MCC**

**Table S3a. Cross-validated AUC (mean ± SD; repeated stratified 10-fold × 5).**

| **Feature set** | **RF** | **SVM** | **XGB** | **LR** |
| --- | --- | --- | --- | --- |
| **Fre** | 0.974±0.011 | 0.971±0.014 | 0.974±0.012 | 0.957±0.016 |
| **GPT** | 0.916±0.026 | 0.906±0.029 | 0.909±0.028 | 0.918±0.024 |
| **DS** | 0.923±0.026 | 0.915±0.026 | 0.914±0.029 | 0.922±0.027 |
| **GPT-Fre** | 0.976±0.013 | 0.970±0.015 | 0.975±0.013 | 0.967±0.014 |
| **DS-Fre** | 0.975±0.014 | 0.970±0.015 | 0.974±0.013 | 0.968±0.013 |
| **Phys** | 0.906±0.024 | 0.909±0.024 | 0.898±0.025 | 0.884±0.031 |
| **k-mer** | 0.932±0.023 | 0.947±0.019 | 0.928±0.020 | 0.912±0.021 |

**Table S3b. Cross-validated MCC (mean ± SD; repeated stratified 10-fold × 5).**

| **Feature set** | **RF** | **SVM** | **XGB** | **LR** |
| --- | --- | --- | --- | --- |
| **Fre** | 0.814±0.056 | 0.826±0.050 | 0.831±0.057 | 0.787±0.059 |
| **GPT** | 0.697±0.075 | 0.693±0.070 | 0.664±0.072 | 0.688±0.064 |
| **DS** | 0.726±0.068 | 0.700±0.065 | 0.705±0.066 | 0.719±0.056 |
| **GPT-Fre** | 0.826±0.064 | 0.815±0.054 | 0.840±0.061 | 0.794±0.058 |
| **DS-Fre** | 0.830±0.056 | 0.820±0.054 | 0.833±0.057 | 0.801±0.057 |
| **Phys** | 0.648±0.075 | 0.658±0.067 | 0.639±0.074 | 0.620±0.070 |
| **k-mer** | 0.723±0.068 | 0.752±0.051 | 0.706±0.063 | 0.684±0.067 |

**Table S4. Shuffled-rules control**

**Table S4. CV accuracy (mean ± SD) for real vs across-peptide-permuted rule vectors.**

| **Model** | **GPT-Fre** | **GPT-Fre (shuffled)** | **DS-Fre** | **DS-Fre (shuffled)** |
| --- | --- | --- | --- | --- |
| **RF** | 0.912±0.033 | 0.909±0.028 | 0.913±0.029 | 0.909±0.026 |
| **SVM** | 0.906±0.027 | 0.894±0.030 | 0.909±0.027 | 0.890±0.029 |
| **XGB** | 0.919±0.031 | 0.915±0.026 | 0.916±0.029 | 0.910±0.028 |
| **LR** | 0.896±0.029 | 0.887±0.030 | 0.900±0.029 | 0.883±0.033 |

**Table S5. Paired significance tests**

**Table S5. Paired t-test and Wilcoxon signed-rank tests across the 50 CV folds (RF).**

| **Comparison (RF, 50 folds)** | **mean A** | **mean B** | **Δ** | **t-test p** | **Wilcoxon p** |
| --- | --- | --- | --- | --- | --- |
| **GPT-Fre vs Fre** | 0.912 | 0.906 | +0.006 | 4.92e-02 | 6.13e-02 |
| **DS-Fre vs Fre** | 0.913 | 0.906 | +0.008 | 2.25e-02 | 3.83e-02 |
| **GPT-Fre vs GPT-Fre(shuffled)** | 0.912 | 0.909 | +0.003 | 3.36e-01 | 5.05e-01 |
| **DS-Fre vs DS-Fre(shuffled)** | 0.913 | 0.909 | +0.005 | 1.66e-01 | 2.19e-01 |
| **GPT(rules) vs Phys** | 0.846 | 0.822 | +0.024 | 8.46e-04 | 2.42e-03 |
| **DS(rules) vs Phys** | 0.860 | 0.822 | +0.038 | 2.32e-07 | 2.81e-06 |
| **GPT(rules) vs k-mer** | 0.846 | 0.860 | -0.014 | 3.20e-02 | 5.48e-02 |
| **DS(rules) vs k-mer** | 0.860 | 0.860 | +0.000 | 9.52e-01 | 8.43e-01 |

**Table S6. Held-out test split**

**Table S6. Metrics on the authors’ original 739/185 held-out split (n = 185).**

| **Model (RF, 739 train / 185 test)** | **ACC** | **AUC** | **F1** | **MCC** |
| --- | --- | --- | --- | --- |
| **RF-Fre** | 0.946 | 0.988 | 0.948 | 0.893 |
| **RF-GPT** | 0.838 | 0.922 | 0.835 | 0.685 |
| **RF-GPT-Fre** | 0.903 | 0.982 | 0.906 | 0.806 |
| **RF-DS** | 0.859 | 0.932 | 0.860 | 0.724 |
| **RF-DS-Fre** | 0.897 | 0.975 | 0.898 | 0.799 |

**Table S7. Low-data learning curve**

**Table S7. RF accuracy (mean ± SD; 20 stratified resamples) at increasing training fractions.**

| **Train fraction** | **~N seq** | **Fre** | **GPT-Fre** | **DS-Fre** |
| --- | --- | --- | --- | --- |
| **0.1** | 92 | 0.876±0.022 | 0.883±0.017 | 0.884±0.022 |
| **0.2** | 184 | 0.890±0.016 | 0.893±0.017 | 0.892±0.018 |
| **0.3** | 277 | 0.892±0.018 | 0.898±0.012 | 0.899±0.013 |
| **0.5** | 462 | 0.897±0.013 | 0.897±0.013 | 0.902±0.013 |
| **0.7** | 646 | 0.903±0.011 | 0.905±0.013 | 0.907±0.014 |

**Table S8. Sequence novelty of the de novo candidates**

**Table S8. Maximum global-alignment identity of each candidate to CPP924, TAT and Penetratin.**

| **ID** | **Length** | **Max identity vs CPP924 (%)** | **Identity vs TAT (%)** | **Identity vs Penetratin (%)** |
| --- | --- | --- | --- | --- |
| **GPT-1** | 18 | 38.9 | 22.2 | 16.7 |
| **GPT-2** | 12 | 53.3 | 25.0 | 18.8 |
| **GPT-3** | 23 | 37.5 | 30.4 | 8.7 |
| **DS-1** | 22 | 40.9 | 27.3 | 18.2 |
| **DS-2** | 18 | 50.0 | 27.8 | 11.1 |
| **DS-3** | 27 | 35.7 | 29.6 | 14.8 |

**Table S9. Generation acceptance rates**

**Table S9. Rejection-sampling acceptance rates over a 100,000-sequence run.**

| **Sampling prior** | **Passed ≥15/20 rules** | **Passed ≥15/20 + prob ≥0.80** |
| --- | --- | --- |
| **CPP-class composition prior** | 30.54% | 28.01% |
| **Uniform amino-acid prior** | 2.05% | 0.27% |

**Table S10. Direct LLM classification vs ML models**

Each of the 185 held-out test peptides (same split as the ML evaluation; 92 CPP / 93 non-CPP) was given directly to a current-version LLM (temperature 0, single zero-shot prompt, no examples), which returned a binary CPP/non-CPP label. Accuracy, F1 and MCC are compared with the authors’ ML models on the identical split (cf. Reviewer 5, follow-up to Comment 7).

**Table S10a. Accuracy, F1 and MCC: direct LLM classification vs ML models (same test set).**

| **Method** | **Accuracy** | **F1** | **MCC** |
| --- | --- | --- | --- |
| **DeepSeek-V3 (deepseek-chat), direct** | 0.773 | 0.716 | 0.592 |
| **GPT-4o (openai/gpt-4o), direct** | 0.854 | 0.832 | 0.731 |
| **RF-Fre** | 0.946 | 0.948 | 0.893 |
| **RF-GPT-Fre** | 0.903 | 0.906 | 0.806 |
| **RF-DS-Fre** | 0.897 | 0.898 | 0.799 |

**Table S10b. Error analysis of the direct-LLM predictions (high precision, low recall).**

| **Direct-LLM model** | **Precision** | **Recall** | **TP** | **TN** | **FP** | **FN** |
| --- | --- | --- | --- | --- | --- | --- |
| **DeepSeek-V3 (deepseek-chat)** | 0.946 | 0.576 | 53 | 90 | 3 | 39 |
| **GPT-4o (openai/gpt-4o)** | 0.971 | 0.728 | 67 | 91 | 2 | 25 |

**Supplementary Figures**

**Figure S1. ROC and precision–recall curves for all model × feature-set combinations, re-exported at high resolution (600 dpi).**

**Figure S2. Cross-validated accuracy (mean ± SD) for every classifier × feature-set combination.**

**Figure S3. Shuffled-rules control: real vs across-peptide-permuted rule vectors for the four classifiers.**

**Figure S4. Low-data learning curve: accuracy of Fre, GPT-Fre and DS-Fre versus training-set fraction.**

**Figure S5. Sequence-novelty analysis of the six de novo candidates.**

**Figure S6. AlphaFold-3 (Model 0) per-residue confidence (pLDDT) of the de novo peptides and reference CPPs.**

**Figure S7. AlphaFold-3 structural-similarity metrics (TM-score and Cα-RMSD) versus TAT and Penetratin.**
